# Presence of Potential Enteropathogenic Bacteria in Cats and Association With Diarrhea in Multicat Households

**DOI:** 10.1111/jvim.70138

**Published:** 2025-05-22

**Authors:** Kirsten Bogedale, Ute Klein‐Richers, Sandra Felten, Michèle Bergmann, Nikola Pantchev, Yury Zablotski, Jan Suchodolski, Kathrin Busch, Stefan Unterer, Katrin Hartmann

**Affiliations:** ^1^ Small Animal Specialist Hospital North Ryde New South Wales Australia; ^2^ LMU Small Animal Clinic Centre for Clinical Veterinary Medicine, LMU Munich Munich Germany; ^3^ Clinic for Small Animal Internal Medicine Vetsuisse Faculty, University of Zurich Zurich Switzerland; ^4^ IDEXX Laboratories Kornwestheim Germany; ^5^ Gastrointestinal Laboratory College of Veterinary Medicine, Texas A&M University College Station Texas USA

**Keywords:** bacterial enteropathy, cattery, fecal penal, feces, feline

## Abstract

**Background:**

Diarrhea in cats is common in multicat households, and fecal panels are frequently used to diagnose potential enteropathogenic bacteria.

**Objectives:**

To evaluate the presence of potential fecal enteropathogenic bacteria and their association with diarrhea in multicat households. The null hypothesis was that potential enteropathogenic bacteria were not related to diarrhea in the cohort.

**Animals:**

In total, 234 cats from 41 privately owned catteries were included.

**Methods:**

In this prospective study, feces were evaluated for consistency according to a visual scoring system (Purina Fecal Score). Scores from 4 to 7 were defined as diarrhea. Quantitative PCR for *Escherichia coli*, *Clostridium perfringens* encoding the *α* toxin gene (*cpa*), *Clostridium perfringens* encoding the enterotoxin gene (*cpe*), *Clostridioides difficile*, *Campylobacter jejuni/coli*, and *Salmonella enterica* was used. Logistic regression was used to evaluate the impact of selected bacteria on diarrheic feces (fecal score ≥ 4). *p* values were corrected for multiple comparisons (*q* values in results). A *q* value < 0.05 was considered to indicate statistical significance.

**Results:**

23/234 cats (9.8%) had diarrhea. None of the selected bacteria were significantly associated with diarrhea (*cpa*: *q* = 0.10, *cpe*: *q* = 0.20, *C. jejuni* : *q* = 0.70). *E. coli* was detected in all tested fecal samples (100%). The associations of *Cl. difficile* (detected in 2.3% of cats), *C. coli* (0%), and *S. enterica* (0.9%) with diarrhea could not be evaluated due to the small sample sizes.

**Conclusions and Clinical Importance:**

Fecal bacteria detected via PCR were not associated with clinical signs of diarrhea in multicat households. These data do not support the use of PCR for fecal bacteria for baseline routine diagnostic work‐up of diarrhea in cats.

**Abbreviations**
*C. jejuni/coli*
: *Campylobacter*
95% CI: 95% confidence intervals
*Cl. difficile*
: *Clostridioides*

*C. difficile*

:
*Clostridium difficile*

*cpa*
:
*Clostridium perfringens* encoding for *α* toxin gene
*cpe*
:
*Clostridium perfringens* encoding for enterotoxin gene
*E. coli*
: *Escherichia*
g: gram
OR: odds ratio
qPCR: quantitative PCR
*S. enterica*
: *Salmonella*
spp.: Species

## 1 Introduction

Diarrhea is a common clinical sign in cats [1]. The risk of having diarrhea is greater in cats living with multiple fellows [2]. Cats in crowded conditions are more likely to harbor intestinal pathogens, and affected cats are prone to infection with more than 1 pathogen [3, 4, 5, 6]. When cats have less space available, the prevalence of intestinal protozoal infections is greater [7]. Additionally, increased stress levels are associated with small enclosure sizes, and crowding stress in groups with multiple cats could have an immunosuppressive effect on the individual animal [8, 9].

A thorough workup of diarrhea includes a detailed history, exclusion of non‐gastrointestinal etiologies, assessment of the absorptive function of the small intestine, and testing for endoparasites and involves both laboratory and imaging diagnostics, as well as tissue sampling [10]. Additionally, many laboratories offer fecal panels that include PCR for viral, bacterial, and protozoal organisms. Currently, most fecal panels combine fecal flotation with antigen testing for endoparasites and cultures and PCR upon request by clinical veterinarians. The interpretation of the results of fecal enteric panels must be performed cautiously, and the detection methods must be considered. In dogs, fecal bacterial culture panels have low diagnostic value [11, 12]. Putative enteropathogenic bacteria are isolated from feces of cats and dogs with and without diarrhea and detected by real‐time PCR at similar rates, but diarrhea is not significantly associated with enteropathogenic infections in cats [13, 14, 15]. In contrast, bacteria invading the intestinal mucosa are associated with gastrointestinal inflammation if they are histologically detected in biopsy samples [16, 17]. Nonetheless, the assessment of suspected pathogenic bacteria in feces is still widely performed in cats suffering from diarrhea. The identification of potentially pathogenic fecal bacteria in cats with diarrhea might prompt general practitioners to prescribe antimicrobials, as is often observed in routine clinical practice. Since there is no national obligation to report the use of antimicrobial drugs in cats, exact numbers are not available. Treatment with antimicrobials without a proper indication is a major concern, as it is associated with several negative consequences, including the development of antibiotic resistance, disruption of the normal microbiota, and the potential for adverse effects on the cat [18, 19, 20].

The primary aim of this study was to evaluate the role of potential enteropathogenic bacteria in diarrhea in cats living in multicat households. The null hypothesis was that the presence of potential enteropathogenic bacteria would not be associated with diarrhea in this cohort. The second aim of this study was to determine the influence of husbandry in catteries on the presence of specific intestinal bacteria. It was hypothesized that crowded housing conditions would be associated with a greater prevalence of potential enteropathogenic bacteria.

## 2 Materials and Methods

### 2.1 Cats

In this prospective study, the owners of breeding catteries were contacted in person at breeding shows or via email and phone. The inclusion criterion for breeding catteries was the housing of at least 5 cats, with 1 being an intact queen. Cats with preexisting conditions related to or treated with medication likely affecting the gastrointestinal tract were excluded. The cats were considered healthy by their owners.

The study was approved by the responsible veterinary authority (Government of Upper Bavaria; reference number 55.2‐1‐54‐2532.2‐14‐13). The owners provided informed consent before participation. The cats and samples included in the present study were also included in other studies, each of which had a distinct research question and therefore an individual analytical approach [21, 22, 23].

All owners completed a questionnaire to obtain information about the cats and their living conditions. The questionnaire was published previously [21]. Evaluating the impact of husbandry conditions on the presence of potential enteropathogenic bacteria was a main focus of this study. Specific factors were as follows: Number of cats per household, space per cat (m^2^), outdoor access (yes/no), raw diet (yes/no), number of toilets ≥ number of cats + 1 (yes/no), disinfection of toilets per month.

A sample of fresh feces from every individual cat was collected at home by the owners, shipped refrigerated in a commercial fecal tube to the investigators immediately, and submitted to a reference laboratory where it was analyzed using a commercially available diagnostic fecal panel by quantitative PCR (qPCR) within 48 h of sample collection. The feces were kept at 4°C until analysis. The remaining feces were stored in commercial microcentrifuge tubes at −80°C. Because 2 organisms of interest, *Escherichia coli* and *Clostridioides difficile*, formerly known as *C. difficile* , were not part of this routine panel, fecal samples were later shipped frozen via express mail and analyzed by qPCR to detect these 2 organisms. Detection of fecal *Cl. difficile* and quantification of fecal *E. coli* were performed on samples from 222 cats.

To determine fecal consistency, every fresh sample was evaluated according to a visual scoring system (Purina Fecal Scoring Chart, Nestlé Purina, St. Louis, USA; Table 1) upon arrival at the clinic by the same veterinary professional. It was considered a modification if feces were evaluated after collection and immediate refrigerated shipping. The scores ranged from 1 to 7. For statistical analyses, scores from 1 to 3 were defined as nondiarrheic consistency, whereas scores from 4 to 7 were defined as diarrhea.

**TABLE 1:** Purina Fecal Scoring Chart, Nestlé Purina, St. Louis, USA, with modifications.

Fecal score	Description of fecal consistency
1	Very hard and dry^a^ *Often expelled as individual pellets* *Requires much effort to expel from the body* *Leaves no surface residue when picked up*
2	Firm, but not hard, pliable^a^ *Segmented appearance* *Leaves little or no surface residue when picked up*
3	Log shaped, moist surface^a^ *Little or no visible segmentation* *Leaves surface residue, but holds form when picked up*
4	Very moist and soggy^a^ *Log shaped* *Leaves surface residue and loses form when picked up*
5	Very moist, but still distinct shape^a^ *Present in piles rather than logs* *Leaves surface residue and loses form when picked up*
6	Has texture, but no defined shape^a^ *Present as piles or spots* *Leaves surface residue when picked up*
7	Watery^a^ *No texture* *Present in flat puddles*

^a^ Characteristics that were evaluated after collection and immediate shipping of feces to the examiners. Characteristics *in italics* were not evaluated due to collection and handling of feces by the cats' owners.

### 2.2 Detection of Intestinal Bacteria

Fecal samples were examined by commercially available qPCR assays to detect *Clostridium perfringens* encoding the *α* toxin gene (*cpa*), *Clostridium perfringens* encoding the enterotoxin gene (*cpe*), *Campylobacter jejuni/coli*, and *Salmonella enterica*. Extraction of fecal DNA and qPCR assays, as well as analysis of the qPCR results, were performed as previously reported [4, 22, 24]. The quantities were reported for *cpa* and *cpe* [25]. Cats were considered positive for *cpa* and *cpe* if the copy number was > 300 000 per gram (g) of feces (Leutenegger et al. Abstract ID‐38 Toxin Quantification of *Clostridium perfringens* is a predictor for diarrhea in dogs and cats. 2012 ACVIM Forum Research Abstracts Program. *Journal of Veterinary Internal Medicine* 26 (2012): 690–822). The DNA extraction method, PCR primers, and qPCR conditions for *E. coli* and *Cl. difficile* used were as previously described by Sung et al. and Werner et al. [24, 26].

### 2.3 Data Analysis

Software R version 4.0.3 (2020‐10‐10, R Foundation for Statistical Computing, Vienna, Austria) and SPSS Statistics version 28.0.1.1 were used.

Descriptive analysis was performed to describe the cats' signalment. The prevalence of diarrhea and of fecal scores indicating diarrhea in the cohort was calculated.

Statistical analysis was performed to analyze the association between the occurrence of diarrhea or husbandry and the detection and quantity of specific bacterial taxa, respectively.

The proportion of diarrheic cats to nondiarrheic cats was compared with a 2‐sample test for the equality of proportions with Yates' continuity correction due to the small sample size. By univariate analysis, the impact of any selected bacteria on diarrheic feces (fecal score ≥ 4) and the impact of husbandry conditions on the detection of selected bacteria were evaluated. Logistic regression was applied. More specifically, univariable logistic regressions estimated the probability of *E. coli* , *cpa*, *cpe*, *Cl. difficile*, *C. jejuni/coli*, and *S. enterica* as binary response variables (0 and 1) by numerous predictors, i.e., presence of diarrhea, number of cats per household, space per cat, outdoor access, raw diet, number of toilets ≥ number of cats + 1, and disinfection of toilets per month. *p* values were corrected for multiple comparisons (q‐values in the results). A *q* value < 0.05 was considered to indicate statistical significance. Multivariate analysis was not performed due to the small sample size.

The prevalence of *E. coli* and *cpa* in the study cohort and in diarrheic and nondiarrheic cats was calculated. Furthermore, the quantitative association of *E. coli* and *cpa* with the presence of diarrhea was assessed. Both organisms were chosen for quantitative assessment due to their high prevalence within the study cohort. The data were tested for normality using the Shapiro–Wilk normality test due to the small sample size. The Mann–Whitney *U* test was applied to determine whether there were any differences in the quantities of the two bacteria between diarrheic and nondiarrheic cats. A *p* value < 0.05 was considered to indicate statistical significance.

The exact 95% confidence intervals (95% CIs) for percentages were calculated by the Clopper–Pearson method.

For visualization purposes, a basic nonparametric bootstrap was applied to estimate confidence limits for the study cohort mean without assuming normality. This approach was combined with box plots to enhance comparability.

## 3 Results

### 3.1 Cats

In total, 239 cats from 41 catteries were included. According to the number of cats, husbandries were categorized as 5 cats (*n* = 14), 6–10 cats (*n* = 52) or > 10 cats (*n* = 168). After excluding 5 animals due to megacolon (*n* = 1), enterectomy years prior (*n* = 1), hyperthyroidism (*n* = 1), and suspected dietary hypersensitivity (*n* = 2), the results of 234 cats from 37 catteries were finally evaluated in the study.

The cats' ages ranged from 2 months to 12 years. Age was not reported by the owners for 3 cats (1%). Half of the cats (*n* = 127, 54%) were up to 2 years of age. One‐quarter of the cats (*n* = 58, 25%) were between 2 and 4 years of age. Twenty‐two (9%) cats were between 4 and 6 years of age, and 12 (5%) cats were between 6 and 8 years of age. Ten cats (4%) were between 8 and 10 years old, and 2 cats (1%) were older than 10 years. The majority of the cats (*n* = 137, 59%) were intact females; 18 cats (7%) were spayed females, 64 cats (27%) were intact males, and 15 cats (6%) were neutered males.

The study cohort consisted of purebred cats of various breeds: British Shorthair (*n* = 57), Bengal (*n* = 51), Birman (*n* = 32), Maine Coon (*n* = 17), Norwegian Forest Cat (*n* = 22), Oriental (*n* = 1), Persian (*n* = 18), Savannah (*n* = 3), Scottish Fold (*n* = 4), Scottish Straight (*n* = 4), Sphynx (*n* = 4), Somali (*n* = 8), Taiga (*n* = 1), Turkish Angora (*n* = 3) and Turkish Van (*n* = 7). The breeds of the 2 cats were not recorded.

### 3.2 Presence of Diarrhea and Potential Enteropathogenic Fecal Bacteria

In total, 23/234 cats (9.8%) had diarrhea: a fecal score of 4 was present for 17 cats (7.3%), a score of 5 was present for 4 cats (1.7%), and a score of 6 was present for 2 cats (0.9%). A fecal score of 7 was not present in any cat. The 23 cats with diarrhea were distributed across 14 catteries. None of the tested bacteria were significantly associated with the presence of diarrhea (Table 2).

**TABLE 2:** Association of detected potential enteropathogenic bacteria in feces with the presence of diarrhea.

Bacterium	Fecal score (number of cats)							Total	%	Non‐diarrhea/diarrhea	*q* value
	1 (23)	2 (146)	3 (42)	4 (17)	5 (4)	6 (2)	7 (0)				
*Escherichia coli*	22	138	41	17	3	1	0	222/222	100	201/21	Not evaluated
*cpa*	20	114	33	16	4	2	0	189/234	80.8	167/22	0.10
*cpe*	3	13	2	0	0	0	0	18/234	7.7	18/0	0.20
*Clostridioides difficile*	1	3	1	0	0	0	0	5/222	2.3	5/0	Not evaluated
*Campylobacter jejuni*	2	5	1	2	0	0	0	10/234	4.3	8/2	0.70
*Campylobacter coli*	0	1	0	0	0	0	0	1/234	0	1/0	Not evaluated
*Salmonella enterica*	1	0	0	1	0	0	0	2/234	0.9	1/1	Not evaluated

*Note:* Cats are grouped according to their fecal scores and detection of bacteria in feces. Scores 1–3 indicate non‐diarrheic fecal consistency, scores 4–7 indicate diarrhea. *Escherichia coli*, *Clostridium perfringens* encoding for *α* toxin gene (*cpa*), *Clostridium perfringens* encoding for enterotoxin gene (*cpe*), *Clostridioides difficile*, *Campylobacter jejuni/coli* and *Salmonella enterica*. Logistic regression was applied. *p* values were corrected for multiple comparisons (*q* value in the results). A *q* value < 0.05 was considered statistically significant. Not evaluated: The presence of *E. coli* was not included in the analysis, since this bacterium was detected in each of the remaining 222 tested samples. The roles of *Cl. difficile*, *C. coli* and *S. enterica* on the presence of diarrhea were not evaluated due to very small sample sizes.

*E. coli* was detected in all tested fecal samples, and 21/222 (9.5%, 95% CI 6.0–14.1) cats had diarrhea. There was no statistically significant difference in the quantity of *E. coli* between diarrheic and nondiarrheic cats (*U* = 1727.00, *Z* = −1.370, *p* = 0.17). Diarrheic cats had a median of 4.6 (95% CI 4.3–5.3, range 3.3–6.7) log_10_ copy numbers of *E. coli* per g of feces. A median of 4.3 (95% CI 4.0–4.5, range 1.4–8.8) log_10_ copy numbers of *E. coli* per g of feces was detected in nondiarrheic cats.

**FIGURE 1:**
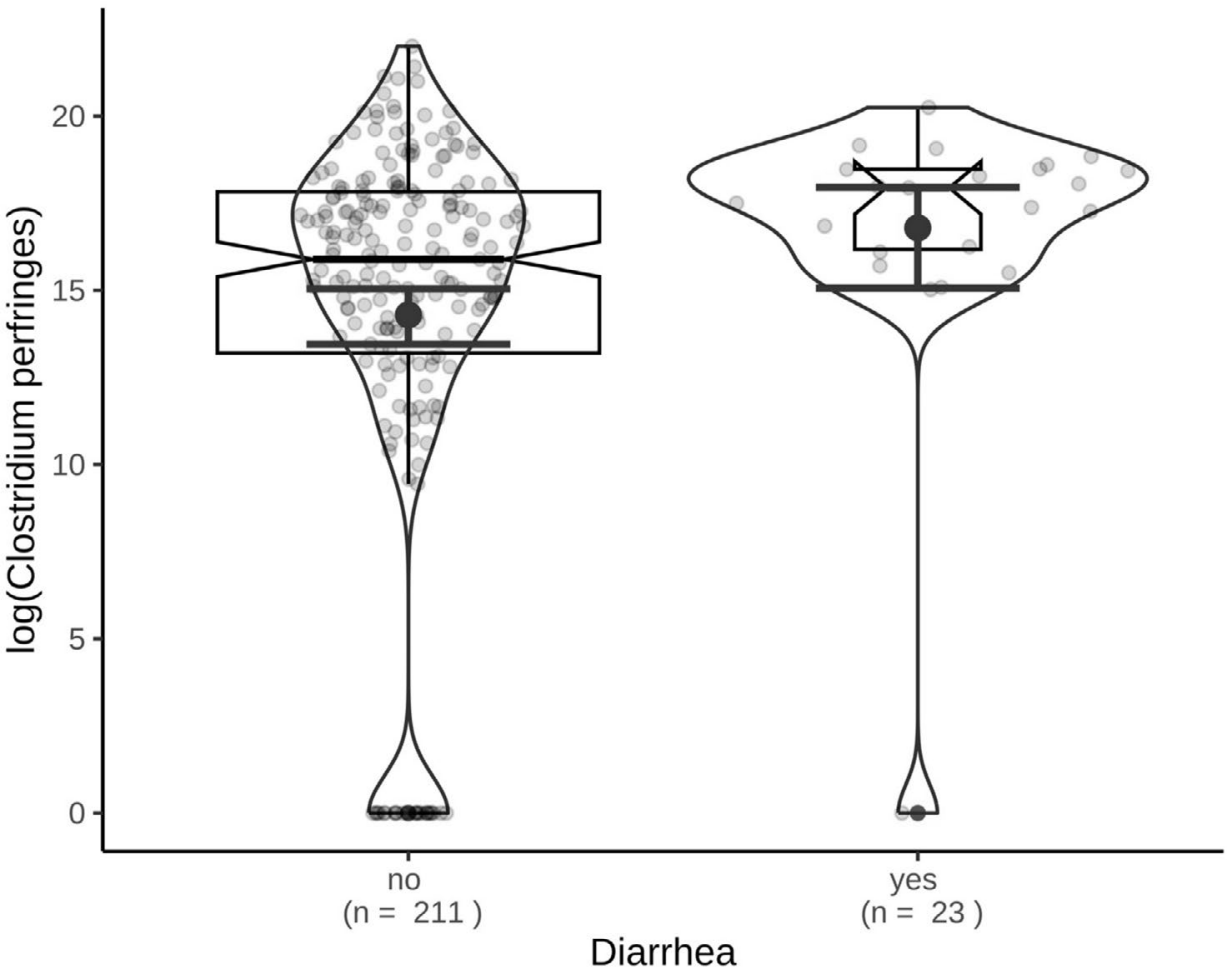
Quantity of *Clostridium perfringens* encoding the *α* toxin gene in log_10_ copy numbers per gram of feces in diarrheic versus nondiarrheic cats. The violin plot shows the distribution of the data. Boxplot with notches indicate the 95% CI for the median. The error bars show the mean and 95% bootstrapped CI for the mean.

*Cpa* was detected in 189/234 (80.8%, 95% CI 75.1–85.6) of the cats. Its presence was not significantly associated with the presence of diarrhea (*q* = 0.10). In cats with diarrhea, the prevalence of *cpa* was 95.7% (95% CI 79.1–99.9), whereas in cats without diarrhea, the prevalence of *cpa* was 79.1% (95% CI 73.0–84.4). There were significant differences in the quantity of *cpa* between the two groups (*U* = 1552.00, *Z* = −2.838, *p* = 0.005; Figure 1). Diarrheic cats had a median of 6.2 × 10^7^ (95% CI 3.2 × 10^7^–1.4 × 10^8^, range 0–6.0 × 10^8^) copy numbers of *cpa* per g of feces. In contrast, a median of 7.9 × 10^6^ (95% CI 5.8 × 10^7^–1.5 × 10^8^, range 0–4 × 10^9^) copy numbers of *cpa* per g of feces was detected in nondiarrheic cats.

### 3.3 Influence of Husbandry on the Detection of Bacteria

The influence of husbandry on the presence of *E. coli* was not analyzed since this bacterium was detected in every tested sample. The number of cats within the household and the space available to each cat were significantly associated with the presence of both *cpa* and *cpe* in feline feces, as shown in Tables 3 and 4. *Cpa* was more likely to be present in cats living in groups of more than 5 individuals. If cats had more than 20 m^2^ per cat at their disposal, they were more likely to be positive for *cpa*. *Cpe* was more likely in cats living in groups of 6 to 10 individuals, but less likely in groups of more than 10 individuals than in groups of 5 cats. *Cpe* was more likely to be present in cats with less than 10 m^2^ per cat at their disposal. The detection of *cpe* was also significantly associated with the number of litter boxes available. Cats with access to at least 1 litter box more than the number of cats in the household were more likely to have *cpe* in their feces than cats who had maximally the same number of toilets as the number of cats. Furthermore, the type of diet fed influenced the detection rate of *cpa* and *cpe*: Cats that were fed a raw meat diet (strictly or as part of a combination diet) were more likely to have *cpa* or *cpe* in their feces than cats that received a commercial diet. It must be noted that, despite a statistical significance by hypothesis testing, the ranges of 95% CIs indicated a less precise estimate, and housing conditions might have had a minor association with the detection of bacteria in the study cohort.

**TABLE 3:** Influences of husbandry on the detection of *Clostridium perfringens* encoding for *α* toxin gene (*cpa*) evaluated by univariate logistic regression.

Predictor	Number of cats	*cpa*‐positive	OR^a^, ^b^	95% CI^b^	*q* value^c^
Number of cats per household			***		**0.008**
5	14	7	—	—	
6–10	52	49	11.8	2.79–49.9	
> 10	168	135	3.32	1.15–9.62	
Space per cat (m^2^)			**		**0.01**
≤ 10	146	120	—	—	
11–≤ 20	54	37	0.46	0.23–0.94	
21–≤ 30	17	17	10.3	0.59–180	
31–≤ 50	10	10	6.43	0.36–114	
> 50	7	7	4.85	0.26–89.4	
Outdoor access					0.90
No	130	107	—	—	
Yes	104	84	0.91	0.46–1.77	
Raw diet			**		**0.03**
No	54	37	—	—	
Yes	180	154	2.63	1.32–5.52	
Number of toilets ≥ number of cats +1					0.70
No	223	183	—	—	
Yes	11	8	0.62	0.16–2.76	
Disinfection of toilets per month					> 0.90
≤ 1	43	35	—	—	
≥ 2	191	156	1.02	0.41–2.30	

*Note:* *q* value < 0.05 was considered as statistically significant are in bold.

^a^ *p < 0.05; **p < 0.01; ***p < 0.001.

^b^ OR = odds ratio; CI = confidence interval.

^c^ False discovery rate correction for multiple testing.

**TABLE 4:** Influence of husbandry on the detection of *Clostridium perfringens* encoding for enterotoxin gene (*cpe*) evaluated by univariate logistic regression.

Predictor	Number of cats	*cpe‐*positive	OR^a^, ^b^	95% CI^b^	*q* value^c^
Number of cats per household			***		**0.008**
5	14	1	—	—	
6–10	52	11	3.13	0.59–67.6	
> 10	168	6	0.46	0.07–9.57	
Space per cat (m^2^)			***		**0.001**
≤ 10	146	11	—	—	
11–≤ 20	54	1	0.31	0.06–1.68	
21–≤ 30	17	0	0.22	0.01–3.97	
31–≤ 50	10	6	16.3	4.21–62.9	
> 50	7	0	0.38	0.02–8.56	
Outdoor access					0.50
No	130	8	—	—	
Yes	104	10	1.57	0.62–4.40	
Raw diet					0.40
No	54	2	—	—	
Yes	180	16	2.24	0.69–16.5	
Number of toilets ≥ number of cats +1			**		**0.03**
No	223	14	—	—	
Yes	11	4	7.13	1.92–26.5	
Disinfection of toilets per month					0.90
≤ 1	43	3	—	—	
≥ 2	91	15	1.12	0.35–5.07	

*Note:* *q* value < 0.05 was considered as statistically significant are in bold.

^a^ *p < 0.05; **p < 0.01; ***p < 0.001.

^b^ OR = odds ratio; CI = confidence interval.

^c^ False discovery rate correction for multiple testing.

*C. jejuni* detection was not significantly influenced by any of the husbandry conditions (Table 5).

**TABLE 5:** Influence of husbandry on the detection of *Campylobacter jejuni* evaluated by univariate logistic regression.

Predictor	Number of cats	*C. jejuni‐*positive	OR^a^, ^b^	95% CI^b^	*q* value^c^
Number of cats per household					> 0.90
5	14	0	—	—	
6–10	52	3	2.29	0.22–23.6	
> 10	168	7	1.72	0.18–16.4	
Space per cat (m^2^)					> 0.90
≤ 10	146	6	—	—	
11–≤ 20	54	2	0.90	0.21–3.92	
21–≤ 30	17	1	1.32	0.19–9.07	
31–≤ 50	10	1	2.04	0.26–15.8	
> 50	7	0	0.49	0.02–13.8	
Outdoor access					
No	130	7	—	—	
Yes	104	3	0.57	0.11–1.93	
Raw diet			*		0.20
No	54	0	—	—	
Yes	180	10	8.84	0.52–150	
Number of toilets ≥ number of cats +1					0.80
No	223	9	—	—	
Yes	11	1	1.90	0.12–14.8	
Disinfection of toilets per month					0.30
≤ 1	43	4	—	—	
≥ 2	191	6	0.36	0.09–1.29	

^a^ *p < 0.05; **p < 0.01; ***p < 0.001.

^b^ OR = odds ratio; CI = confidence interval.

^c^ False discovery rate correction for multiple testing.

The influence of husbandry on the presence of *Cl. difficile*, *C. coli*, and *S. enterica* was not evaluated due to small sample sizes.

## 4 Discussion

The aim of this prospective study was to assess the association between potential enteropathogenic bacteria and fecal consistency in cats in multicat households. None of the bacteria tested in this study were associated with the occurrence of diarrhea in this cohort.

*E. coli* is considered part of the physiologic intestinal microbiome in cats and can be isolated from both healthy and diseased cats [13, 27]. It was found in all of the cats, and the quantity of *E. coli* present in feces was not associated with the presence of diarrhea in this cohort. This finding mirrors previous studies in which the abundance of *E. coli* does not differ significantly between healthy cats and cats with acute or chronic diarrhea [28]. Cats with chronic enteropathy have an increased abundance of *E. coli* compared to healthy control cats [24]. However, it is important to highlight that in most cats in our study, the number of *E. coli* was within the provided reference range, and according to previous research, there is considerable overlap between healthy and diseased individuals [24]. Fecal PCR for *E. coli* might therefore likely not be a helpful diagnostic tool for diarrheic cats. To further evaluate the role of *E. coli* in individual cases, the classification of *E. coli* type and molecular or histological applications should be considered instead [17].

*Cpa* was present in 80.8% of the fecal samples, and its detection in the study group was not associated with the presence of diarrhea (*q* = 0.10). There is a significant association of *cpa* with diarrhea in cattery cats (*p* = 0.03) [22]. In the present study, several univariate models were tested, so the data were corrected for the false discovery rate. The applied q‐value as a measure of significance included correction for multiple testing to avoid false‐positive results [29, 30]. This statistical adaptation might explain why there was no reported association between the detection of *cpa* and diarrhea in the present study group. Cats in larger living spaces were more likely to have *cpa*. This finding initially appeared contradictory since we hypothesized that cats in more crowded housing conditions would have a greater prevalence of bacteria. But the *α* toxin gene is present in every *C. perfringens* strain, and thus, its presence likely does not indicate virulence [13]. However, the amount of *cpa* varied in this cohort: Cats with diarrhea had a significantly greater median copy number of *cpa* than nondiarrheic cats, which could be explained by potential imbalances in the intestinal microbiome of the cats. An overgrowth of *C. perfringens* is presumably connected to intestinal dysbiosis in chronic enteropathies [31]. Increased proportions of *C. perfringens* are observed in dogs with acute diarrhea [32, 33]. As more detailed information about the diarrhea of each individual (e.g., duration of diarrhea) was not available in the present study, cats were not grouped as acutely or chronically diarrheic, but dysbiosis might be present in both scenarios. The quantity of *C. perfringens* does not differ between healthy cats and cats with chronic or acute diarrhea [28]. Unlike in dogs, *C. perfringens* therefore might not play an important role in gastrointestinal diseases in cats [28]. Although diarrheic cats had significantly greater amounts of *cpa*, median *cpa* values in both diarrheic and nondiarrheic cats were greater than the suggested cutoff (by Leutenegger et al. Abstract ID‐38 Toxin Quantification of *Clostridium perfringens* is a predictor for diarrhea in dogs and cats. 2012 ACVIM Forum Research Abstracts Program. *Journal of Veterinary Internal Medicine* 26 (2012): 690–822). The type of diet might have influenced the *cpa* results in the study group, and the diet is linked to the catteries. For that reason, the type of diet fed in a specific cattery might have had more influence than the space available to the cats. In the present study, cats were more likely to have *cpa* if they were fed a partial or completely raw meat diet than if they were fed a commercial diet. *C. perfringens* ' detection can be increased by a high‐protein and high‐fat diet (raw meat), as observed in dogs [34]. Despite the statistical significance by hypothesis testing, according to the present data, the CIs were wide and mostly overlapped between the two groups, indicating a less precise estimate. Thus, the significant differences must be interpreted cautiously with regard to clinical relevance and applicability. *C. perfringens* and *cpa* were not associated with diarrhea in the study group and their abundance might be influenced by factors other than fecal consistency such as the cat's diet.

*Cpe* was detected in 7.7% of the study group. A prevalence of 2.0% in healthy cats and 4.1% in cats with diarrhea has been reported [13]. Interestingly, in the present study, *cpe* was detected only in nondiarrheic fecal samples and therefore was not associated with diarrhea in the study group. However, proportions between nondiarrheic and diarrheic cats were not balanced in the present study, with the majority of cats having normal fecal consistency. Since *cpe* has a low overall prevalence in cats, it was more likely to be detected in a larger group, which was the group of nondiarrheic cats in the present study. The membrane‐active *C. perfringens* enterotoxin is associated with several gastrointestinal diseases in humans [35]. Dogs with acute diarrhea have a greater abundance of *cpe* than healthy individuals in 1 study, but *cpe* is reported in 33.7% of healthy individuals [36]. Additionally, in chronic diarrhea in dogs, *C. perfringens* enterotoxin genes are unlikely to influence pathogenesis [12]. The role of *cpe* in diarrhea in cats remains unclear.

*Cl. difficile* was present in 5 cats with normal fecal consistency but was not detected in cats with diarrhea. The overall prevalence was 2.3%, which was lower than that reported in previous studies (7.1%–38.1% in cats); however, these studies were performed in a hospital environment [37, 38, 39, 40]. In the present study, feces were collected at home from mostly healthy individuals. This factor might have influenced the detection rate, as healthy outpatient cats are less likely to yield *Cl. difficile* than their inpatient fellows [38]. In contrast to previous studies, the present detection of *Cl. difficile* was determined by the more sensitive PCR instead of bacterial culture, which possibly influenced the detection rate [13]. An association between the presence of *Cl. difficile* and feline intestinal disease is reported based on the detection of toxins in feces [41]. These results are questioned by other studies on feline fecal samples where toxin activity is not detected in toxicogenic strains, and nontoxicogenic types of *Cl. difficile* are far more prevalent in cats than toxicogenic isolates [42, 43]. A greater abundance of *Cl. difficile* is considered a consequence of intestinal dysbiosis caused by a lack of secondary bile acids in feline and canine chronic enteropathies and has been recently shown to be associated with a lack of the major bile acid converter *Clostridium hiranonis* in dogs [26, 31]. However, as mentioned before, classification into chronic or acute gastrointestinal disease was not performed in the present study group. The low detection rate within the cohort and detection in only nondiarrheic cats suggest that *Cl. difficile* likely might not have played a role in diarrhea for the cats in this study.

*C. jejuni* was present in 4.3% of the cats within the study group and is reported at similar rates in both healthy cats and those with diarrhea [44, 45, 46, 47, 48]. Two/10 positive cats had diarrhea, and the presence of *C. jejuni* was not associated with the presence of diarrhea, supporting previous results [46, 49]. No risk factors for the presence of *C. jejuni* were identified either in the present study or previously [45, 47]. One cat with normal fecal consistency was positive for *C. coli* . The prevalence of this disease is less than 1% in both healthy and diarrheic cats [45, 46, 47, 48]. Little is known about the role of *C. coli* in cats. The affected cat in the present study was fed a raw meat diet, but more specific information was not available. *C. coli* contaminates raw meat poultry samples as well as raw meat pet foods [50, 51]. It is possible that the cat might have been infected by its diet. In human medicine, campylobacteriosis is the most commonly reported zoonosis, with *C. jejuni* and *C. coli* being the most common etiologic agents [52, 53]. *C. jejuni* isolates from humans and their pets overlap, suggesting a possible risk of infection of humans by their pets [47, 54]. The risk for human infection by *C. jejuni* /*coli* is not associated with contact with animals in general but is increased by exposure to animals with diarrhea [55]. However, because of the low overall prevalence of *C. jejuni* in cats, this species is considered to be of limited importance for human infections, and the threat of cats for human health is rated low [45, 47, 54]. The present study is consistent with previous findings that *C. jejuni* /*coli* has minor relevance for gastrointestinal health in cats.

*S. enterica* was detected in 1 cat with diarrhea and 1 cat without diarrhea. The reported prevalence in cats is generally low, and clinically healthy cats can shed *Salmonella* species (spp.) [13, 56, 57, 58]. Thus, the presence of *S. enterica* in feline feces does not necessarily imply that it is the reason for diarrhea. Raw meat diets are potential sources of infection for cats and dogs [59, 60, 61, 62]. In the present study, 1 cat was fed a combination of commercial and raw meat diets. Whether its diet was the origin of infection remains unknown. The cat lived in a multicat household, but *S. enterica* was not detected in the fecal samples of the companion cats. Cats can shed *Salmonella* spp. for 3–6 and up to 12 weeks after recovery from acute illness [57, 63]. Over the course of infection, shedding becomes intermittent and might be reactivated by circumstances such as stress or crowding [57]. Living conditions in the multicat household might have triggered shedding in the 2 individuals in the study. In human medicine, salmonellosis is the second most reported zoonosis [53]. Although food is the major source of infection, cases of salmonellosis after contact with pets have been reported [64, 65, 66]. The handling and feeding of raw diets to pets is also hypothesized to be a possible source of zoonotic transmission to humans [60, 67]. Even though the 2 *S. enterica* ‐positive cats might be considered otherwise asymptomatic carriers, caution must be taken in handling the cats' diets and cleaning the cats' litter boxes.

Although no associations between the studied bacteria and diarrhea were detected, potentially zoonotic bacteria were detected in the study group. Fecal panels might be helpful in assessing the risk of zoonotic infection in vulnerable households. Another area of application of bacterial PCR of feces might be in the field of fecal microbiota transplants to detect appropriate fecal donors.

There were some limitations to this study. More detailed clinical information on the cats was lacking. Furthermore, participating cats were chosen by their owners, and not all cats within each cattery were included. In diarrheic cats, more specific details, for example, duration of signs, defecation frequency, involvement of the small or large intestine, or detailed information about the cats' diets, were not available. Since the clinical sign of diarrhea can be a result of multiple factors, for example, sensitivity to food ingredients, dysbiosis etc., future studies should take more details of the cats' histories and diagnostic work‐up into account and ideally standardize outer influences. Information about husbandry was provided by the breeders via a questionnaire and could not be verified. Exclusion of cats based on previous medication or medical conditions affecting the gastrointestinal tract and causing diarrhea, respectively, relied on the clinician's expertise. We appreciate any concern that this criterion can result in a selection bias and possibly impair proper duplication of this study. Cats with diarrhea and controls were not matched, and cats with nondiarrheic feces were overrepresented; therefore, there might have been a statistical bias. To avoid over‐ and under‐interpretation, future studies should focus on pairing cases with controls. The fecal score was determined after immediate shipping. Both collection and transport might have influenced the fecal consistency, thus changing the resulting score. Since the present study used a subset of original data that was initially collected to answer another scientific question, an a priori power analysis with respect to the present research question was not performed. Some selected bacteria were present in few cats, and thus, the small sample sizes limited the statistical evaluation.

In conclusion, these data do not support the use of PCR for fecal bacteria in the baseline routine diagnostic work‐up of diarrhea in cats.

## Disclosure

Authors declare no off‐label use of antimicrobials.

## Ethics Statement

Institutional Animal Care and Use Committee approval was granted for this study by the Government of Upper Bavaria; reference number 55.2‐1‐54‐2532.2‐14‐13. Authors declare human ethics approval was not needed.

## Conflicts of Interest

Nikola Pantchev is employed at IDEXX Laboratories. IDEXX played no role in the study design, in the collection and interpretation of data, or in the decision to submit the manuscript for publication. Jan Suchodolski is an employee of the Gastrointestinal Laboratory at Texas A&M University, which offers gastrointestinal function and microbiome testing on a fee‐for‐service basis. The other authors declare no conflicts of interest.
